# Piano practice with emphasis on left hand for right handers: Developing pedagogical strategies based on motor control perspectives

**DOI:** 10.3389/fpsyg.2023.1124508

**Published:** 2023-02-14

**Authors:** Jinrui Pang, Shan Zhao, Yilin Wang, Qian Wang, Qun Fang

**Affiliations:** ^1^School of Music, Qingdao University, Qingdao, China; ^2^School of Physical Education, Qingdao University, Qingdao, China; ^3^College of Arts, Beijing Language and Culture University, Beijing, China

**Keywords:** piano education, bimanual control, behavioral performance, neural plasticity, handedness

## Introduction

Piano play involves a series of bimanual movements, thus utilizing both hands fluently is fundamental for a pianist (Furuya and Altenmüller, 2013). Skillful performance requires a high level of precise control over right and left hands. After years of intensive practice, pianists are able to move both hands with great automaticity (Kilincer et al., 2019).

Over the past few decades, piano play has raised an increasing interest among neuroscientists. Pianists provide an ideal model to investigate plastic changes at both behavioral and neural levels (Münte et al., 2002). With the wide application of neuroimaging techniques, researchers are able to study in the neural mechanisms underlying bimanual coordination. Research on piano performance has contributed insights into brain functioning (Globerson and Nelken, 2013). However, efforts of enhancing piano education based on neuroscience evidence remain limited. In fact, the same issue has been brought to notice in the field of physical education. In a recent study, researchers called for attention to asymmetry of interlimb transfer, a typical phenomenon in motor learning and control, which could be a promising pedagogical strategy to enhance sport skill acquisition (Fang et al., 2022).

Based on neural evidence and findings of bimanual control, the current study proposed an opinion that particular attention should be given to the left-hand practice to enhance piano learning for right handers. The improved piano performance and bimanual coordination imply behavioral changes and neural adaptations associated with long-term training, which may provide rationales of highlighting the left-hand practice. Further discussions focus on pedagogical suggestions from a motor control perspective. It is our purpose to provide evidence-based insights into effective approaches to facilitate piano learning and teaching.

## Behavioral changes associated with piano learning

One of the noticeable features of proficient piano performance is the increased independence between left and right hands. A study compared pianists with amateurs when playing two excerpts. Although the excerpts consisted of same notes which should be played by both hands at the same time, the pianist group performed with distinct differences between both hands in dexterous control over timing and force of keystrokes (Kim et al., 2021). The greater hand independence is also displayed as less interference between hands. Research that investigated muscular activities in piano play identified smaller co-activations of hand and finger muscles in experts than amateurs (Furuya et al., 2010, 2011). The findings can be substantiated by subsequent study in mirror movements of pianists and non-piano controls during manual tasks (Chieffo et al., 2016). Mirror movement refers to unintended movements of homologous muscles contralateral to the voluntarily active ones (Schott and Wyke, 1981). The occurrence of mirror movements in healthy individuals suggests challenges in motor control due to task complexity and fatigue (Arányi and Rösler, 2002). While the participants performing finger tapping task as fast as they could, electromyography (EMG) recorded mirror movements in the control group but not in the pianist group. The evidence suggests that the long-term piano experience may induce a strong inhibitory control over involuntary hand movements (Chieffo et al., 2016).

Another prominent change associated with piano skill proficiency is the reduced motor laterality between dominant and non-dominant hands. Hand difference is effective in differentiating pianists from amateurs (Kim et al., 2021). Compared with the controls, musicians are characterized by symmetric motor performance and left-hand superiority (Gärtner et al., 2013). In a reaching accuracy task, piano experts outperformed non-piano controls in terms of left-hand performance, whereas no significant difference was identified between the experts and the controls in task performance by right hand. Thus, a smaller between-hand difference was identified in the experts compared with the controls (Kilincer et al., 2019). Consistent findings were also reported in a study which included a pegboard test and a finger tapping test (Chieffo et al., 2016). Pianists showed similar performance of left and right hands, whereas novices completed the tasks with superior performance on the right hand. A lower asymmetry index (a measure of performance difference between left and right hands) was found in the pianist group than that in the control group, suggesting the tendency of less motor asymmetry associated with increased piano proficiency.

The reduced motor laterality is also evident in hand preference. Pianists tend to use left hand more often than controls when reaching targets in their frontal space, indicating altered pattern of hand selection associated with piano experience (Kilincer et al., 2019). According to the evidence from both piano play and bimanual tasks, pianists are characterized by balanced hand preference and reduced manual asymmetry due to enhanced left-hand performance.

## Neural adaptations associated with piano learning

Applications of neuroimaging techniques provide insights into neural mechanisms underlying bimanual control. In a positron emission tomography (PET) study, stronger activations were identified in premotor cortex and supplementary motor area during bimanual finger movements, suggesting an active role of the cortical regions in bimanual coordination (Sadato et al., 1997). Premotor cortex is responsible for planning and organizing voluntary movements (Sira and Mateer, 2014). Research has shown particular involvement of premotor cortex in the early phase of motor learning to set up cognitive strategies and motor program of complex movements (Filippi et al., 2010; Gryga et al., 2012). Once the motor sequence is established, premotor cortex becomes less activated in motor control (Andres et al., 1999; Lafleur et al., 2002). Corpus callosum consists of white matter tracts that connect left and right hemispheres. Due to the primary function of interhemispheric communication, corpus callosum is important for bimanual performance (Serrien et al., 2001). Based on magnetic resonance imaging (MRI) technique, researchers found a connection between structural organization of the corpus callosum and capacity of bimanual coordination (Muetzel et al., 2008). The higher factorial anisotropy, a measure of structural properties of the corpus callosum, suggests increased interhemispheric connectivity which contributes to skilled performance in bimanual tasks. Johansen-Berg et al. (2007) examined integrity of white matter in corpus callosum by diffusions MRI. Variability in white matter integrity in callosal pathways was found to correlate with individual differences in bimanual performance.

Piano practice induces structural and functional changes in the brain. Hyde et al. (2009) investigated influences of piano training on brain structure by comparing young children (Age: 6.32 ± 0.82 years) who regularly attended piano lessons with their counterparts (Age: 5.90 ± 0.54 years) who did not receive any instrumental training. While no difference between the groups was found in the beginning of the study, significant differences were identified in primary motor cortex, primary auditory cortex, and corpus callosum after 15 months, indicating the differential brain development due to the piano training in early childhood.

Hemispheric lateralization is considered an outcome of evolution, which allows superior cognitive capacity and motor performance in more lateralized individuals (Rogers, 2000, 2002). For example, as a representation of lateralized hemispheric function, handedness enables individuals to complete a difficult task with the stronger and more dexterous hand (Corballis, 2019). However, neural evidence indicates reduced hemispheric asymmetry associated with piano training. In an MRI study, pianists were characterized by symmetrical size of the primary motor cortex while the non-pianist controls indicated left-larger-than-right asymmetry (Amunts et al., 1997). Houdayer et al. (2016) identified balanced interhemispheric interactions along with improved piano skill proficiency. Specifically, ipsilateral silent period (ISP), a measure of callosal transmission, was asymmetric at the baseline, with the left hemisphere characterized by longer duration than the right hemisphere. Increased ISP duration was identified in the right hemisphere after 10 days of piano training, leading to symmetrical ISP between hemispheres in the post-test. The findings were consistent with the research which identified more symmetrical motor cortical representation in pianists (Chieffo et al., 2016). For the right-handed controls with no musical experience, cortical representation of the dominant hand muscles was larger than that of the non-dominant hand muscles. Pianists, however, indicated larger cortical representation of the non-dominant hand muscles, leading to the reduced asymmetry in motor representation. Hemispheric asymmetry has been considered to favor the dominant hand performance in unimanual tasks. In contrast, bimanual performance such as piano play demands a comparable level of skills between hands. The long-term piano experience induces symmetrical neural functioning and brain structure which, in turn, may facilitate piano learning and performance.

## Pedagogical implications based on motor control perspectives

For right handers, left hand is considered weaker and slower in functional performance (Kim et al., 2021). Left-hand skills need more attention in practice because optimal performance in a challenging bimanual task can be achieved only if hand differences are minimal (Kopiez et al., 2006). To familiarize learners with left-hand play, a commonly used strategy is the part-whole approach with initial practice on single hand before training both hands together. However, effectiveness of the teaching approach may be limited in that the mechanisms between unimanual and bimanual control are similar but not identical (Van Vugt and Altenmüller, 2019). Unimanual training on each hand may hardly result in complete transfer of learning to bimanual performance. Therefore, bimanual practice with particular emphasis on the left hand is worth further notice in piano education.

Given the rationales of highlighting left-hand practice in piano education, pedagogical strategies can be developed by taking advantage of bimanual coordination. Research has shown a greater influence of the dominant left hemisphere on the non-dominant left hand than that of the non-dominant right hemisphere on the dominant right hand (Kagerer, 2016). The asymmetrical influence implies greater interference on the left-hand performance. Indeed, when task demands on bimanual coordination are high, right handers tend to prioritize right-hand performance at the cost of left-hand performance (Kopiez et al., 2006).

Cognitive related approach was often used to reduce interference on the left hand in bimanual performance (Peters and Schwartz, 1989). Researchers have shown that more attention is paid to the hand playing melody (Parncutt et al., 1999). Accordingly, to raise attention to the left hand during piano play, it is reasonable to practice the musical score in which melody is performed by left hand. This pedagogical suggestion can be particularly useful for novices who often face difficulty in controlling both hands at the same time. With left hand playing the melody part, novices may reduce right-hand interference by giving more attention to the left-hand performance.

Movement tempo could be another critical consideration in piano education. Given that more attention would be paid to the limb moving at a higher frequency (Panzer et al., 2021), the faster moving limb has a significant impact on the slower moving limb (Kennedy et al., 2016, 2017). Improved stability and accuracy have been identified with the faster moving hand regardless of handedness (Summers et al., 1993; Peper et al., 1995; Panzer et al., 2021). In order to improve left-hand performance of the right handers, a reasonable strategy is to choose the musical score with left hand playing at a higher tempo than right hand.

Attention plays an essential role in learning and performance. Increases in attentional effort, mediated by top-down mechanisms, is necessary to maintain or recover performance under challenging circumstances (Sarter et al., 2006). Top-down attentional modulation primarily involves prefrontal, parietal, and limbic areas which represent essential cognitive activities in task performance such as selective attention, divided attention, working memory, and motivation (Mcdowd, 2007). Additionally, bottom-up mechanism can be activated when an individual becomes highly focused from a less focused state. The ascending reticular activating system, involving reticulothalamocortical pathway and extrathlamic pathways, regulates the switch in mental states (Mcdowd, 2007). In the early stage of learning to play a musical score, multiple factors have to be attended, with more attentional resources recruited in learning and performance.

It is also important to raise awareness of higher neural efficiency associated with enhanced proficiency. Compared with initial stage of learning, autonomous stage after intensive repetitions and practice is characterized by reduced activation of the attention system without compromising performance. An fMRI study identified smaller activation of cortical areas in pianists than controls when performing a complex finger movement task, which provided evidence for the reduced cortical activations in pianists (Krings et al., 2000). The sequenced movement suggests programmed performance during piano play. Motor program refers to a set of stored muscle commands ready for action at any time (Schmidt, 1975). Once the program has been established, performance can be generated in an efficient manner. The features of programmed performance are in line with Hebbian mechanisms which account for strengthened synaptic connections through mere repetitions to achieve effortless performance (Makino et al., 2016). This process is classified as unsupervised learning which is dominant at the later stages of motor learning. The strengthened connections between coactive neurons build neural circuits which generate a stable, reproducible activity pattern in support of autonomous performance (Fiete et al., 2010). Therefore, pedagogical strategies that direct attentional efforts to the non-dominant left hand are necessary to reduce interference by the dominant right hand in the initial stage of learning a novel musical score. With practice and improved proficiency, attention is expected to decline as performance becomes autonomous.

## Conclusions

The current study proposed an opinion as to enhancing piano education based on the understandings of bimanual control. Piano experience over a long period of time induces plastic changes in behavioral performance as well as brain structure and functioning. Compared with novices and non-piano controls, pianists are characterized by reduced motor laterality and increased hand independence. Neural adaptations associated with the piano experience indicate less hemispheric asymmetry which facilitates bimanual coordination in piano play. The changes at behavioral and neural levels provide insights into pedagogical strategies. Left-hand practice should be highlighted in piano teaching and learning by (i) practicing the musical scores with left hand playing the melody parts, and (ii) practicing the musical scores with left hand playing at the higher tempo. The pedagogical suggestions were reached by means of qualitative analysis on the existing literature. Empirical evidence is needed in future research to testify efficacy of the pedagogical suggestions in practice.

## Author contributions

QW and QF: conceptualization. JP and SZ: draft preparation. YW, QW, and QF: validation. YW: review and editing. All authors collaborated in preparing the manuscript and approved the submitted version.
